# Determination and clinical features of bone pseudoprogression in metastatic breast cancer

**DOI:** 10.3389/fonc.2025.1643179

**Published:** 2025-09-25

**Authors:** Ting Xu, Yikun Ma, Lili Zhang, Shiyi Li, Min Dong, Yuan Yuan

**Affiliations:** ^1^ Department of Oncology, The Affiliated Cancer Hospital of Nanjing Medical University, Nanjing, China; ^2^ Departments of Radiology, Jiangsu Cancer Hospital, Jiangsu Institute of Cancer Research, The Affiliated Cancer Hospital of Nanjing Medical University, Nanjing, China; ^3^ Department of Chemotherapy, The Affiliated Cancer Hospital of Nanjing Medical University, Jiangsu Cancer Hospital, Jiangsu Institute of Cancer Research, Nanjing, China

**Keywords:** breast cancer, bone metastasis, pseudoprogression, osteoblastic metastasis, alkaline phosphatase

## Abstract

**Objective:**

Bone is one of the most common sites of metastasis for breast cancer. The classification of new osteoblastic lesions as progressive disease is currently controversial. Computed tomography (CT), specifically the bone window setting, is the most frequently utilized for evaluating treatment efficacy in bone metastatic breast cancer (MBC). In this study, we aimed to assess the clinical features and significance associated with bone pseudoprogression.

**Methods:**

This retrospective analysis was conducted among twenty-three MBC patients with new osteoblastic lesions during the first-line systemic therapy in Jiangsu Cancer Hospital from January 2018 to December 2023. After assessing treatment response every two cycles by CT (bone window) at least twice, we identified no disease progression in participants, thus defining new osteoblastic lesions as bone pseudoprogression. Participants continued treatment until explicit disease progression was observed (extraosseous disease progression or progressive lysis on bone lesions). Pretreatment baseline and follow-up alkaline phosphatase (ALP) levels were analyzed separately at the times of bone progression and pseudoprogression in the same patient.

**Results:**

The spine (78.2%) was the predominant metastatic site. The median time to the appearance of bone pseudoprogression after treatment was 1.73 months (95% CI: 1.42-2.04). Furthermore, the median interval between bone pseudoprogression and disease progression was 14.27 months (95% CI: 12.18-16.35). No significant difference in the interval was observed between HER2-positive and HER2-negative MBC patients (15.83 months versus 14.23 months, p=0.79). Compared to the occurrence of disease progression, the levels of ALP decreased or stabilized at pseudoprogression in the same patient. The difference in ΔALP between pseudoprogression and progression was statistically significant (-20U/L vs. 33 U/L, p< 0.001).

**Conclusions:**

Osteoblastic new lesions detected using CT (bone window) may be considered bone pseudoprogression, which predominantly occurs in the early stages of treatment. ALP serves as a biomarker for differentiating pseudoprogression from disease progression on CT (bone window) in patients with bone metastasis. Clinicians should exercise caution regarding the appearance of new osteoblastic lesions in patients not exhibiting extraosseous disease progression or progressive lysis on bone lesions.

## 1 Introduction

Bone is one of the most common sites of metastasis for breast cancer. Approximately 80% of patients with metastatic breast cancer (MBC) develop bone metastases over the disease course (1). Complications of bone metastasis include bone pain, spinal cord compression, pathologic fractures, and hypercalcemia, all of which can significantly impair the quality of life and the survival rate of patients (2). Furthermore, breast cancer with bone metastases is often considered incurable. The optimal treatment is aimed that delay bone metastasis progression, alleviate pain, prevent skeletal-related events (SREs), and enhance the quality of life (3).

Currently, no universally recognized standard exists for evaluating the efficacy of treatment for bone metastases in breast cancer, which are often deemed “unmeasurable” (4). Bone scans (BSs) are commonly utilized for identifying metastases at an earlier stage and monitoring whole-body bone metastasis (5). However, because of low specificity, BSs can produce false-positive results. Despite improvements in extraosseous lesions and decreased tumor markers among MBC patients during systemic therapy, new osteoblastic lesions, confirmed by CT, paradoxically appeared on BSs and improved on subsequent scans (6, 7). Professor Zhang et al. initially defined this phenomenon as bone pseudoprogression (8). The bone pseudoprogression can be misinterpreted as disease progression, which may precipitate premature changes in systemic therapy regimens, impacting both treatment strategies selection and the duration of effective drugs use.

Consequently, the comprehensive application of various imaging techniques to accurately distinguish between bone pseudoprogression and disease progression is important in evaluating the efficacy of treatment for bone metastasis in breast cancer. Fluorodeoxyglucose F 18 ([^18^F]FDG) positron emission tomography/computed tomography (^18^F-FDG PET/CT), CT, and magnetic resonance imaging (MRI) are conventional approaches for assessing bone metastases. CT scans, particularly bone window scans, significantly contribute to evaluating treatment responses (9), and surpass BSs in detecting bone disease (10). Hence, we defined bone pseudoprogression as the appearance of new osteoblastic lesions on CT (bone window) in the absence of extraosseous disease progression, and no additional new lesions observed on subsequent CT (bone window) at least twice.

Previous studies have endeavored to differentiate bone pseudoprogression from disease progression using biochemical bone markers, such as osteocalcin, cross-linked carboxy-terminal telopeptide of type I collagen (ICTP), and serum tartrate-resistant acid phosphatase 5b (TRACP5b) (11). However, these markers have not yet been clinically implemented. Among the various biochemical bone markers, alkaline phosphatase (ALP) is clinically accessible and instrumental in assessing responses to systemic therapy for bone metastasis (12).

In this study, we aimed to assess the clinical features and significance associated with bone pseudoprogression, and to determine if ALP can effectively distinguish between bone pseudoprogression and disease progression in clinical practice.

## 2 Materials and methods

### 2.1 Data source and study population

We collected data from twenty-three MBC patients who received first-line treatment in Jiangsu Cancer Hospital from January 2018 to December 2023. The patients were characterized by the presence of new osteoblastic lesions in the absence of extraosseous disease progression during the first-line treatment. All patients received the standard first-line treatment recommended by the guidelines (13).

### 2.2 Procedures and assessment

All the patients continued treatment until definitive disease progression was observed (extraosseous disease progression or progressive lysis on bone lesions). Extraosseous disease was routinely assessed at baseline and every two cycles by CT or MRI, in accordance with the Response Evaluation Criteria in Solid Tumors (RECIST 1.1) (14). CT (bone window) was performed at baseline and every two cycles to evaluate bone lesions.

Bone pseudoprogression was defined as the appearance of new osteoblastic lesions on CT (bone window) without extraosseous disease progression, and no additional new lesions on subsequent CT (at least twice).

Serum ALP levels were routinely measured during the cycle of systemic therapy. ALP was measured using an enzyme method (Roche Automatic Analyzer Cobas8000 c702; Roche, Germany). Patients were divided into two groups based on the presence or absence of previous bone metastatic lesions. For those with a history of bone metastasis, baseline and follow-up ALP levels were analyzed separately at the time of bone metastasis and bone pseudoprogression. The interval between examination of CT (bone window) and ALP was less than 10 days. The difference between baseline and follow-up ALP was denoted as ΔALP; ΔALP = follow-up ALP minus baseline ALP; ΔALP ratio = “ΔALP”/”baseline ALP” × 100 (%); “increased ALP” was defined as an increase in ΔALP ratio of more than 10% of baseline; “Decreased ALP” was defined as a decrease in ΔALP ratio of more than 10% of baseline; “Stable ALP” was defined as the remainder, being neither increased ALP nor decreased ALP (12).

### 2.3 Statistical analysis

Statistical analyses were conducted using R Studio. The Kaplan–Meier curves were employed for analyzing the median interval between bone pseudoprogression and disease progression, and stratified log-rank tests were utilized to obtain *P* values between subgroups. ΔALP were compared statistically using the Mann–Whitney U test. *P* value<0.05 was considered indicative of statistical significance.

## 3 Results

### 3.1 Patient characteristics

**Figure 1:**
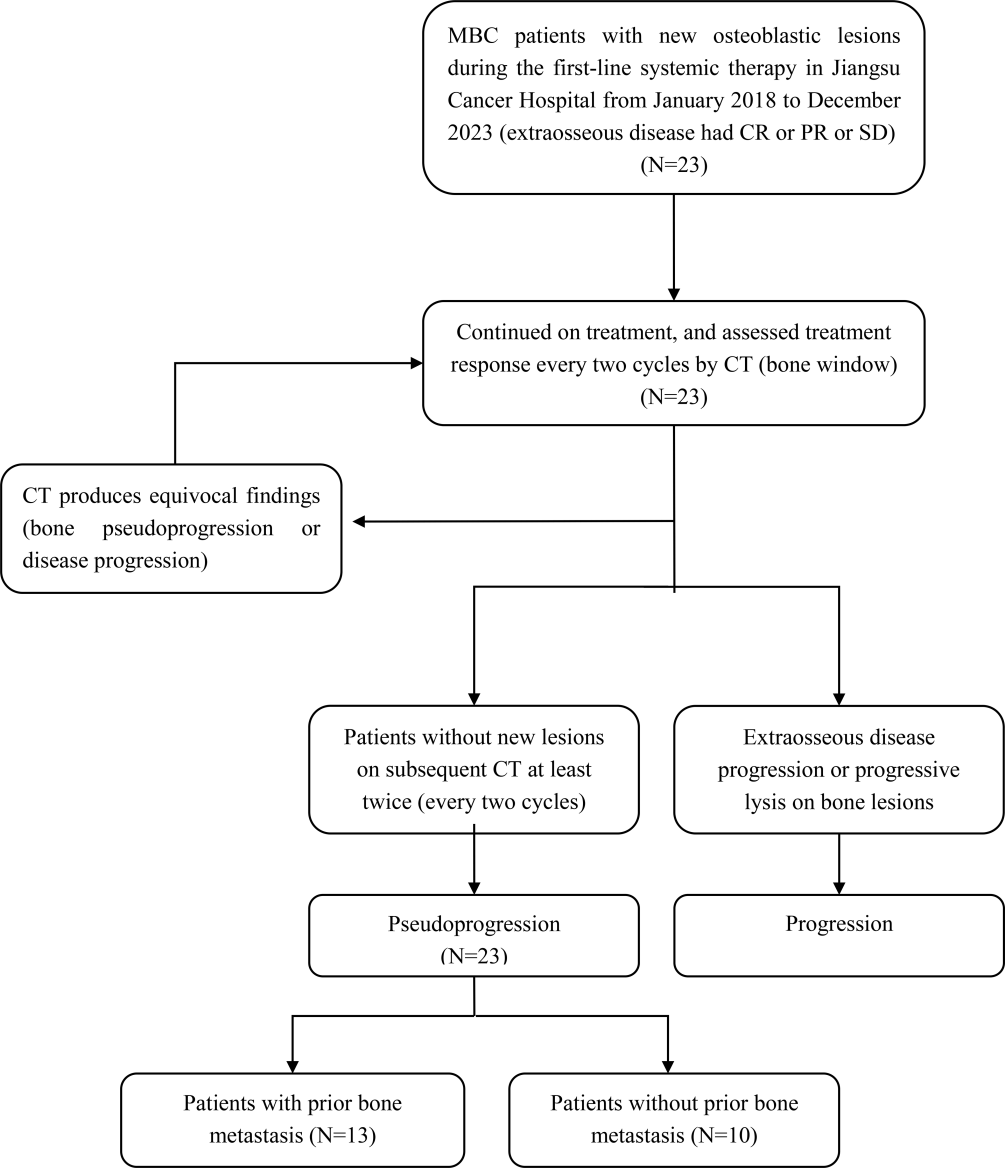
Flow chart of patients inclusion. MBC, metastatic breast cancer; CR, complete response; PR, partial response; SD, stable disease; CT, Computed tomography.

This retrospective analysis was conducted among twenty-three MBC patients who developed new osteoblastic lesions during the first-line systemic therapy, without extraosseous disease progression. All patients continued their treatment regimens and assessed bone lesions every two cycles by CT (bone window). Additionally, CT or MRI was used to assess extraosseous disease every two cycles. As a result, extraosseous lesions of these patients had partial response (PR) or stable disease (SD) and no additional new lesions on subsequent CT (bone window) were included in this study (**Figure 1**).

Baseline characteristics are summarized in **Table 1**. The median age was 52 years (range, 36–70 years). The represented breast cancer molecular subtypes included HER2‐positive (14 of 23, 60.9%), HR‐positive/HER2‐negative (8 of 23, 34.8%), and triple‐negative breast cancer (1 of 23, 4.3%). Among them, 52.2% of patients had visceral metastasis, and 60.9% of these patients had pre-existing metastatic bone lesions. All patients received standard adjuvant and advanced first-line therapy based on different molecular subtypes, and no one received radiotherapy for bone lesions. Nearly all patients (91.3%) received bone-modifying agents, either previously or concurrently, including 78.3% of patients treated with zoledronic acid and 13.0% of patients treated with denosumab.

**Table 1:** Baseline patient characteristics.

Characteristics	Number of patients (%) (N=23)
Age, years, median (range)	52(36-70)
ECOG performance status	
0	5(21.7)
1	18(78.3)
Histological subtype	
Invasive ductal	14(60.9)
Lobular	2(8.7)
Other or not reported	7(30.4)
Molecular subtype	
HER2-positive	14(60.9)
HR-positive/HER2-negative	8(34.8)
Triple negative	1(4.3)
TNM stage at initial diagnosis of cancer	
I	1(4.3)
II	11(47.8)
III	6(26.1)
IV	5(21.7)
Metastatic organ sites	
Bone	23(100)
Lung	5(21.7)
Liver	7(30.4)
Brain	3(13.0)
Lymph nodes	14(60.9)
Chest wall recurrence	1(4.3)
Pleura	3(13.0)
Visceral metastasis	
No	11(47.8)
Yes	12(52.2)
Bone-only metastasis	
No	20(87.0)
Yes	3(13.0)
Prior bone metastasis	
No	10(39.1)
Yes	13(60.9)
Number of metastatic organ sites	
≤3	18(78.3)
>3	5(21.7)
Prior radiotherapy	
No	23(100)
Yes	0
First-line therapy regimen	
Chemotherapy	19(82.6)
Endocrine therapy(including CDK4/6i)	8(34.8)
HER2-targeted therapy	14(60.8)
Prior or concomitant bone-modifying agent	
Zoledronic acid	18(78.3)
Denosumab	3(13.0)
None	2(8.7)

### 3.2 Characteristics of bone metastatic lesions

Whether pre-existing bone metastases or new osteoblastic metastases, the predominant metastatic site was the spine (69.2% or 78.2%), particularly the thoracic and lumbar spine, followed by the pelvis (53.8% or 60.9%), ribs (7.7% or 17.4%), and long bones(7.7% or 4.3%). In addition, 17.4% of new osteoblastic metastases were presented in the sternum and 8.7% in the scapula compared to pre-existing osteolytic and mixed bone metastases (without bone lesions in the sternum and scapula). 61.5% and 73.9% patients had more than two pre-existing and new osteoblastic bone lesions, respectively. The characteristics of the bone metastatic lesions are summarized in **Table 2**.

**Table 2:** Characteristics of bone metastatic lesions.

Characteristics of bone metastatic lesions	Number of patients (%)	
	Pre-existing bone metastatic lesions (N = 13)	New osteoblastic lesions (N = 23)
Type of lesion		
Osteosclerotic	0	23(100)
Osteolytic	6(46.2)	–
Mixed	7(53.8)	–
Sites of lesions on skeleton		
Ribs	1(7.7)	4(17.4)
Long bones	1 (7.7)	1 (4.3)
Pelvis	7 (53.8)	14 (60.9)
Sternum	0	4(17.4)
Scapula	0	2(8.7)
Spine	9(69.2)	18(78.2)
Cervical vertebra	2(15.4)	3(13.0)
Thoracic vertebra	6(46.2)	16(69.6)
Lumbar vertebra	6(46.2)	13(56.5)
Sacral vertebra	7(53.8)	5(21.7)
Total number of new lesions		
1–2	6(46.2)	6(26.1)
>2	8(61.5)	17(73.9)

### 3.3 Effects of new osteoblastic lesions on efficacy of treatment

**Figure 2:**
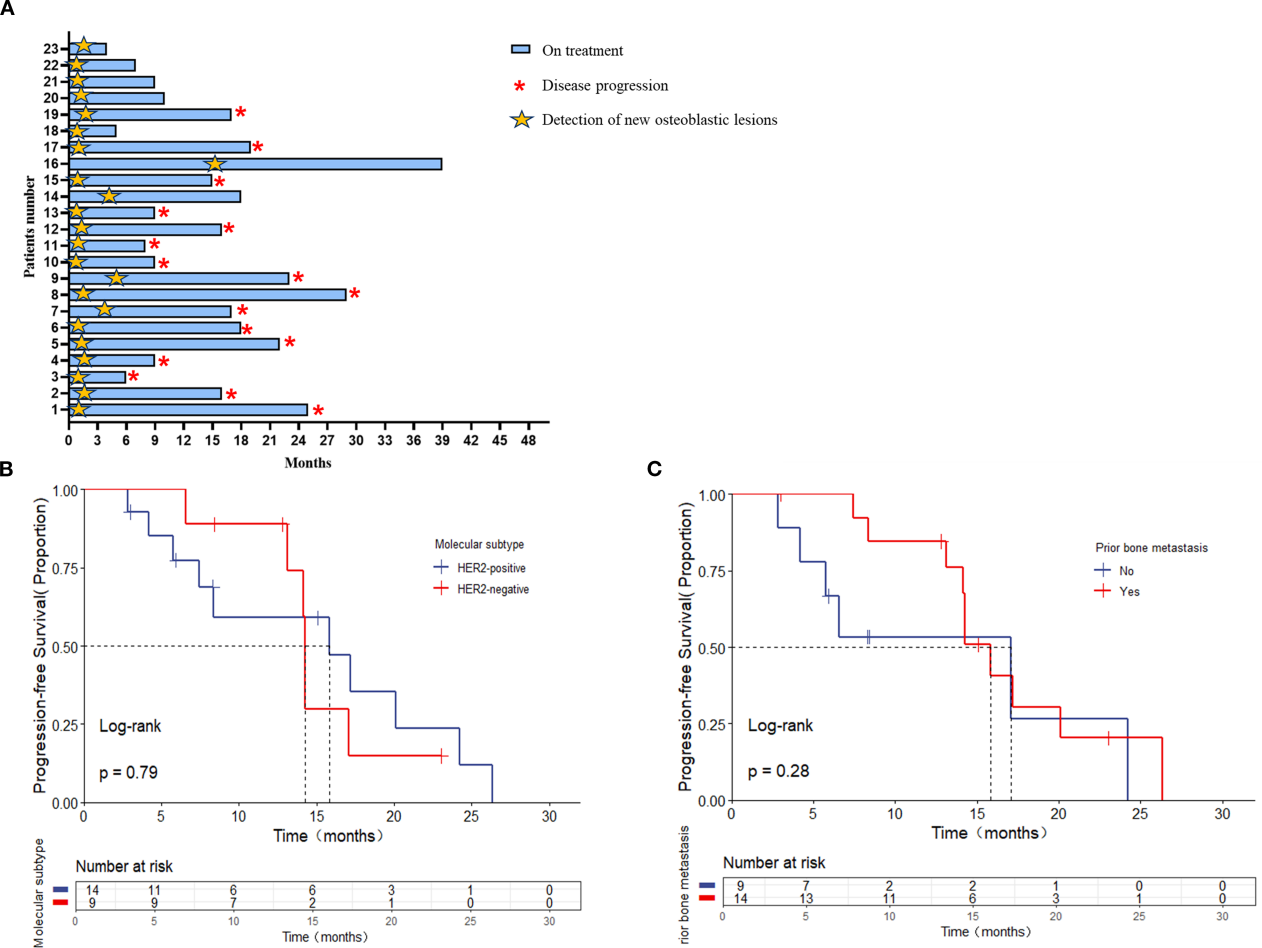
**(A)** The interval among treatment and bone pseudoprogression and disease progression (months); the median interval between bone pseudoprogression and disease progression according to **(B)** molecular subtypes, **(C)** prior bone metastasis.

The median follow-up was 23.0 months (range, 2.8-24.3 months) as of the data cutoff date in February 2024. At that point, seven patients (30.4%) were still receiving the first-line treatment, thirteen patients (56.5%) had discontinued treatment due to extraosseous disease progression, and three patients (13.0%) had experienced progression of bone lesions (**Figure 2A**).

The median time to the appearance of bone pseudoprogression after treatment was 1.73 months (range, 0.57-15.67 months). Among them, nineteen patients (82.6%) with MBC showed bone pseudoprogression within the first 3 months after treatment (**Figure 2A**).

**Figure 3:**
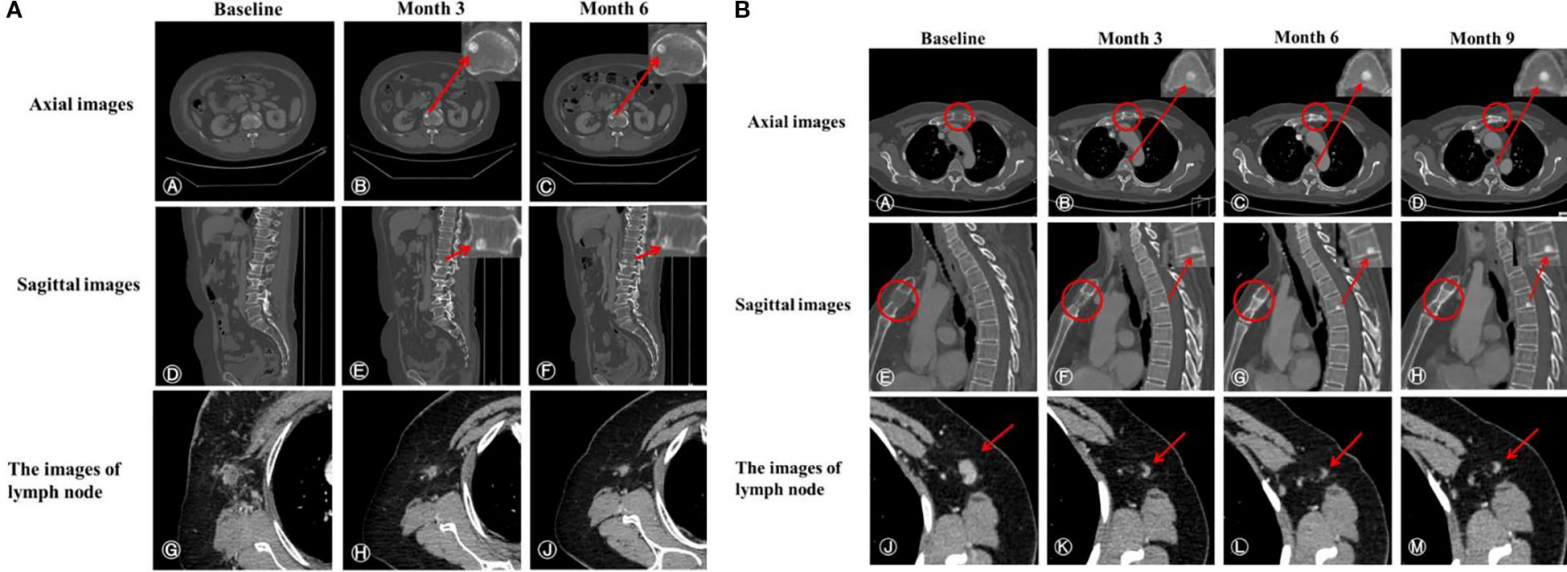
**(A)** CT scans of a patient demonstrating bone pseudoprogression. **(A, D)** Pretreatment axial and sagittal CT images (bone window); **(B, E)** Three-month follow-up reveals a new, well-demarcated, round high-density lesion at the first lumbar vertebra’s inferior edge; **(C, F)** Six-month evaluation shows a sclerotic nodule of similar extent with slightly decreased density; **(G)** Pre-treatment image of an enlarged right axillary lymph node (short diameter 16 mm) with heterogeneous density and indistinct fat margins; **(H, J)** Post-treatment images at three months (short diameter 9 mm) and six months (short diameter 7 mm) demonstrate continuous lymph node regression. **(B)** CT scans of a patient with previous osteolytic metastases demonstrating bone pseudoprogression. **(A, E)** Pre-treatment axial and sagittal bone window images reveal osteolytic metastasis in the sternum. **(B, F)** Three months post-treatment, CT images show a new sclerotic nodule at the lower edge of T4 with clear borders and sclerosis of the original osteolytic metastasis in the sternum. **(C, G)** Six months post-treatment, the sclerotic nodule and the sternal osteolytic metastasis show increased sclerosis and repaired. **(D, H)** Nine months post-treatment, lesion stabilization is observed. **(J)** Pre-treatment left axillary lymph node enlargement (short diameter 10 mm). **(K–M)** Post-treatment regression of left axillary lymph nodes (short diameter 3 mm).

In addition, the median interval between bone pseudoprogression and disease progression was 14.27 months. For instance, a 65-year-old patient, diagnosed with hormone receptor-negative (HR-negative) and human epidermal growth factor receptor 2-positive (HER2-positive) MBC, received trastuzumab, pertuzumab, paclitaxel liposome and carboplatin as first-line therapy. The baseline CT scan revealed multiple metastases in supraclavicular and mediastinal lymph nodes and CT (bone window) presented no metastasis lesions on bone (**Figures 3A** A, D, G). Following two cycles of treatment, lymph node metastases regressed compared to the previous ones, but a new round-like high-density shadow with clear boundaries at the lower edge of the first lumbar vertebra (**Figures 3A** B, E). We considered the sclerotic nodule as bone pseudoprogression and continued the treatment regimen. Subsequent post-treatment CT scans showed no new bone metastases, and the previous osteoblastic lesion had decreased in density (**Figures 3A** C, F), while extraosseous lesions exhibited partial response (PR) (**Figures 3A** H, J). After 9.53 months, the patient remained on treatment without evidence of disease progression (**Figure 3A**). Additionally, we observed bone pseudoprogression in patients with previous osteolytic metastases (**Figure 3B**). The patient’s original osteolytic metastasis in the sternum exhibited increased sclerotization compared to baseline during the first-line treatment, whereas the CT bone window revealed a new, rounded hyper dense lesion at the lower margin of the T4. We maintained the treatment strategy and the subsequent CT follow-up three months later showed that the original sternal osteolytic metastases had progressed to further sclerotization and repair, the osteogenic metastases remained stable, and no new bone metastatic lesions were detected. And the enlarged lymph nodes in the left axilla had significantly regressed.

Besides, stratified by different molecular subtypes, the interval between bone pseudoprogression and disease progression was 15.83 and 14.23 months in HER2-positive and HER2-negative, respectively [*p* = 0.79, (**Figure 2B**). When comparing patients with or without prior bone metastasis, there was no statistically significant difference in the interval, although the group without prior bone metastasis had a longer interval time [17.07 versus 15.83 months, *p* = 0.28 (**Figure 2C**)].

### 3.4 ALP for distinguishing pseudoprogression from disease progression

Thirteen patients previously exhibited bone metastasis (including osteolytic or mixed lesions). Baseline and follow-up ALP levels were analyzed separately at the time of bone metastasis and bone pseudoprogression in the same patient.

**Figure 4:**
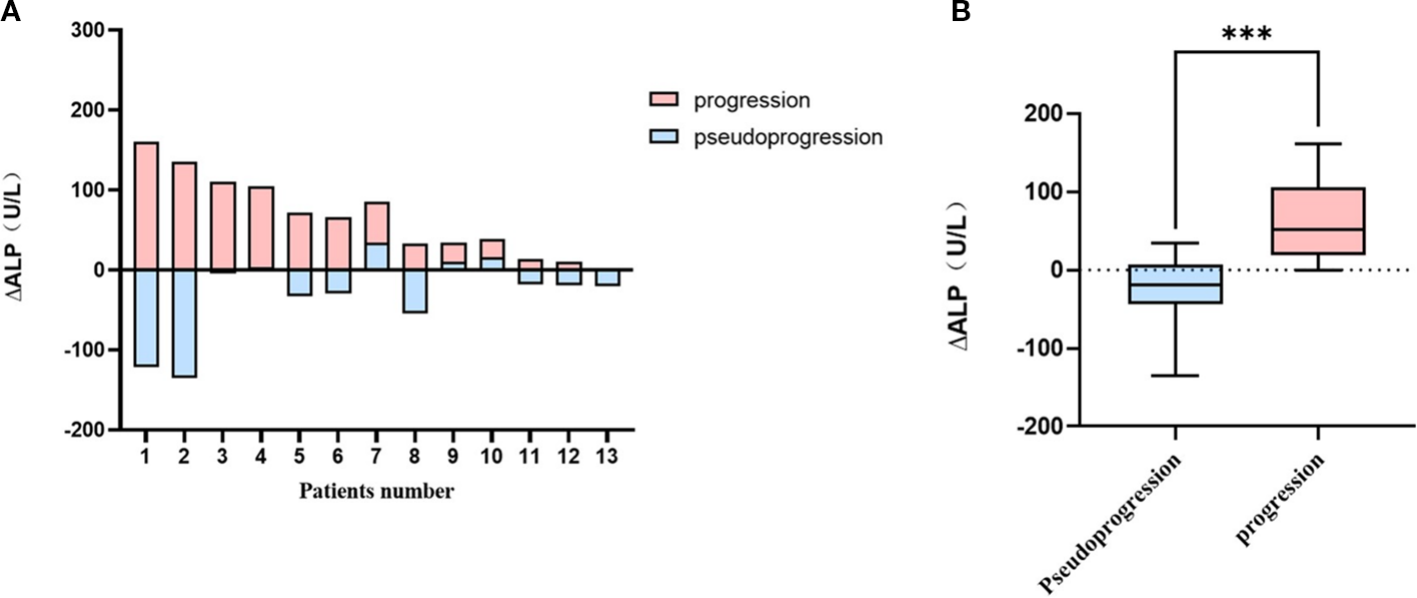
Comparison graphs for ΔALP between the time of pseudoprogression and progression in patients with prior bone lesions **(A)** each patients (N=13), **(B)** in pseudoprogression and progression groups. ***P<0.001.

The median pretreatment and post-treatment ALP levels in the time of progression were 78 (range, 47–140) and 107(range, 65–301) IU/L, respectively, while those in the event of pseudoprogression were 102 (range, 47–301) and 94 (range, 48–193) IU/L, respectively. The incidence of stable or decreased ALP was higher in the times of pseudoprogression than progression (10/13, 76.9% vs. 1/13, 7.7%). In addition, 61.5% of patients experienced a decrease in ALP levels exceeding 10% during pseudoprogression, whereas no patients in the progression group did. **Figure 4A** illustrates the direct changes in ALP levels in the same patients experiencing pseudoprogression and disease progression. ΔALP was significantly different between the events of progression and pseudoprogression (33 vs -20 U/L *p<* 0.001; **Figure 4B**).

## 4 Discussion

Whether new osteoblastic lesions are defined as progression is currently controversial. In this study, we find that new osteoblastic lesions may represent bone pseudoprogression in the absence of extraosseous disease progression, and evaluated the clinical characteristics of bone pseudoprogression and the change in ALP in each patient was informative for differentiating pseudoprogression from disease progression.

BSs are commonly utilized for identifying metastases at an earlier stage. However, the false-positive result observed in BSs may mislead clinical judgment (9). In other words, even if the treatment is effective, the uptake of tracers may increase in the short term. As demonstrated by Song ST et al., BS can only serve as the routine initial screening for bone metastasis. It cannot diagnose bone metastasis nor evaluate the effect of drug therapy (15).In this study, considering that CT scans are superior to BSs for detecting bone disease (10), and CT(bone window)is the most frequently utilized for assessing treatment efficacy in bone metastatic breast cancer. We used CT (bone window) to assess bone metastatic lesions. However, no general consensus has been reached on the optimal imaging methods for monitoring the bone metastasis of breast cancer, and various imaging modalities have advantages and limitations (16). BSs may identify metastases at an earlier stage. However, it has some disadvantages, such as low specificity, no indication of osteoblastic or osteolytic lesions, and no indication of bone destruction from breast cancer bone metastases (17). CT is preferable for assessing bone morphology, whereas MRI is superior for detecting early marrow infiltration and the influences of bone metastases on surrounding tissues (10).18F-FDG PET/CT can demonstrate changes in metabolic activity both pre-and post-treatment (18), however, this method is not as intuitive as CT scanning of bone; Thus, it is not routinely recommended in clinical work and the diagnostic value for breast cancer patients with bone metastases needs to be further demonstrated. In our opinion, the best imaging modality to assess accurate response in bone metastasis is a combination of various imaging methods, and CT bone window allows us to compare the density and size change of the same bone metastasis site more visually before and after treatment.

The definition of bone pseudoprogression was first documented by Professor Zhang in HR+/HER2-MBC patients who received first-line CDK4/6i plus endocrine therapy (8). To the best of our knowledge, all published articles about bone pseudoprogression in breast cancer have involved HR-positive patients who developed bone pseudoprogression during or after CDK4/6i plus endocrine therapy (8, 19, 20). Consequently, some scholars attribute this phenomenon to the utilities of CDK4/6i combined with endocrine therapy. Compared to earlier findings, we observed bone pseudoprogression not only in HR+/HER2-MBC patients treated with CDK4/6i, but also in HER2-positive and TNBC patients, who received standard first-line treatment as recommended by the guidelines. Therefore, we believed there was no direct connection between bone pseudoprogression and CDK4/6i combined with endocrine therapy. In previous studies, bone pseudoprogression has been reported in HR+/HER2-MBC patients, we speculate that this may be due to HR+/HER2- patients account for 65%–70% of breast cancer patients (21). In addition, bone pseudoprogression was observed in patients both with and without previous bone metastasis (**Figure 3**). Therefore, we believed that new osteoblastic lesions represented bone pseudoprogression rather than disease progression when extraosseous lesions remained stable, regardless of whether the patients had a previous combination of osteolytic or mixed metastases.

A prospective study indicated that 75% of patients with MBC exhibited bone pseudoprogression during the first 3 months after treatment (22). Therefore, we defined the patients who appeared osteoblastic lesions, but no additional lesions on subsequent CT at least twice (every two cycles) as bone pseudoprogression in our study. Consistent with these findings, our study demonstrate that bone pseudoprogression predominantly occurs early in the course of treatment, particularly at the initial CT (bone window) evaluation post-therapy (30.4%). After the occurrence of bone pseudoprogression, all patients in our study continued their treatment regimens, with a median interval of 14.27 months between bone pseudoprogression and disease progression. In clinical practice, when a patient develops progression without initiating subsequent treatment, the disease will rapidly progress in a short period of time. In our study, despite the development of new osteoblastic lesions, these lesions remained stable or even improved when the original regimen was maintained. From our point of view, the appearance of new osteoblastic lesions was not considered to be the progression of the disease. Conversely, the emergence of these lesions, indicative of osteoblastic repair, suggested that patients were responsive to therapy, thus warranting the continuation of the original treatment protocol.

Beyond reparative osteogenesis, numerous preclinical studies indicate that the osteogenic niche may initially facilitate early bone metastatic colonization (23, 24). During the early stages of breast cancer bone metastasis, stromal cells surrounding bone-colonizing tumor cells exhibit heightened osteogenic activity, forming specialized microenvironments through E-cadherin/N-cadherin-mediated heterotypic adheres junctions. This osteogenic niche activates Ca^2+^ signaling and mTOR signaling, collectively promoting metastatic progression (23). Critically, our clinical observations of bone pseudoprogression do not contradict but rather complement these findings, revealing the dual dynamic role of bone microenvironments: while osteogenic niches support early metastatic seeding, effective systemic therapy (e.g., endocrine/targeted agents) reprograms mesenchymal stromal cells within these niches toward reparative osteogenesis. This phenotypic shift manifests radiographically as new sclerotic lesions characteristic of bone pseudoprogression. Notably, micrometastases may remain dormant within osteogenic niches for extended periods before triggering the vicious cycle of macrometastatic growth, creating therapeutic opportunities for interception (24). A phase III clinical trial on postmenopausal endocrine-resistant breast cancer(76% of patients with bone metastases) revealed an increase in progression-free survival when everolimus was used in combination with exemestane, indicating that the mTOR inhibitor may efficiently inhibit the progression of metastatic colonization in bone (25). Furthermore, preclinical studies demonstrate that osteoblast-derived Jagged1 promotes both bone metastatic seeding and chemoresistance (26), but anti-Jagged1 therapy (e.g., clone 15D11) in combination with chemotherapy effectively nullifies this resistance mechanism, resulting in superior suppression of bone metastases. These findings position Jagged1 inhibition as a promising strategy for clinical development in both established bone metastases and prevention of early-stage relapse.

Bone formation markers such as ALP, osteocalcin, and ICTP are increased in patients with bone metastases (12). Koizumi et al. reported that an obvious increase in ICTP indicated disease progression, while a slight change in ICTP indicated bone pseudoprogression (27). Besides, the combined use of SQBSI and TRACP5b allows for the differentiation of genuine disease progression from bone pseudoprogression, as has been demonstrated (11). ALP is localized in the membrane of osteoblasts and, thus, represents the activity of osteoblast (28). In a previous study, the “change in ALP” was identified as a useful serologic marker for differentiating pseudoprogression from disease progression in breast cancer patients with bone metastasis (12). In the progressive metastatic bone lesion, osteoclasts and osteoblasts tend to increase, which results in the formation of new destructive bone lesions (29). Consequently, elevated levels of ALP were observed in patients with disease progression, while no significant change or even a decrease in ALP levels was noted in patients who responded to effective treatment. Our findings are consistent with those from earlier studies, the incidence of stable or decreased ALP was higher in the time of pseudoprogression than progression (10/13, 76.9% vs. 1/13, 7.7%).In breast cancer, a significant decrease in serum ALP activity has been observed post-surgery, which suggested that the primary cancer cells may contribute to increased ALP levels (30). However, in our study, we compared ALP levels in the same patient at the time of occurrence of bone pseudoprogression and during the period of only bone lesions progression. The results suggested that assessing response to systemic therapy for bone metastasis should consider the change in ALP levels during therapy.

There were still a few limitations in this study, including being a single-center retrospective study with a small sample size. Although Bone is one of the most common sites of metastasis for breast cancer, osteoblastic metastases in breast cancer are relatively rare. Due to the small sample size, we could not determine the incidence of osteoblastic metastases from breast cancer. Additionally, because of the retrospective study design, evaluation was possible only for serum ALP, which was routinely obtained and it could not be assessed whether other markers could distinguish bone pseudoprogression from disease progression in clinical practice. To overcome these limitations, large prospective studies with comparative and combinatorial analyses using other markers are needed to explore these results further.

## 5 Conclusion

Regardless of whether there were prior osteolytic or mixed bone metastases, the presence of new osteoblastic lesions in the absence of extraosseous disease progression may consider as bone pseudoprogression, which occurs predominantly in the early stages of treatment. Clinicians should exercise caution regarding the emergence of osteoblastic metastases and avoid premature discontinuation of effective treatment due to misjudgments. Decrease or stabilization in ALP levels is recognized as a biomarker indicating the potential for bone pseudoprogression.

## Data Availability

The original contributions presented in the study are included in the article/supplementary material. Further inquiries can be directed to the corresponding authors.
